# Antidiarrheal activity of methanol extract of *Sophora tonkinensis* in mice and spasmolytic effect on smooth muscle contraction of isolated jejunum in rabbits

**DOI:** 10.1080/13880209.2019.1645701

**Published:** 2019-08-22

**Authors:** Yangyou Li, Jing Li, Xin Liu, Jianwu Zhang, Xue Mei, Rudan Zheng, Wei Chen, Qian Zheng, Shangjie Zhong

**Affiliations:** a Animal Experimental Center, North Sichuan Medical College, Nanchong City, China;; b Department of Histology and Embryology, North Sichuan Medical College, Nanchong City, China;; c School of Pharmacy, North Sichuan Medical College, Nanchong City, China;; d Institute of Medicine, North Sichuan Medical College, Nanchong City, China;; e Department of Pathophysiology in School of Basic Medical Science, North Sichuan Medical College, Nanchong City, China;; f Function Center in School of Basic Medical Science, North Sichuan Medical College, Nanchong City, China;; g Department of Clinical Medicine of Chinese and Western Medicine, North Sichuan Medical College, Nanchong City, China

**Keywords:** *Sophora tonkinensis*, antidiarrheal, spasmolytic

## Abstract

**Context:** In China, the herb *Sophora tonkinensis* Gagnep. (Fabaceae, ST) (Committee of National Pharmacopeia. 2015) exhibits anti-inflammatory, antitumor, and antiviral effects. However, to date, there have been few studies on its gastrointestinal effect.

**Objective:** The gastrointestinal effect of the methanol extract of ST rhizome (STR) was evaluated.

**Materials and methods:** Study was conducted from February to December 2018. *In vivo*, antidiarrheal activity of STR (125, 250 and 500 mg/kg; orally) in castor oil-induced diarrheal mice was studied. *In vitro*, the effects of STR (0.01–10 mg/mL) on the isolated tissue preparations of rabbit jejunum were also investigated, the rabbit jejunum stripes were pre-contracted with Ach (10^−5 ^M), K^+^ (60 mM) and tested in the presence of STR, the possible spasmolytic effect was analyzed in the pretreatment of the jejunum preparations with STR or verapamil in Ca^2+^-free high-K^+^ (60 mM) solution containing EDTA.

**Results:** STR (125, 250 and 500 mg/kg) exhibited antidiarrheal activity. STR (0.01–10 mg/mL) completely relaxed spontaneously contracting, Ach (10^−5 ^M) and high K^+^ (60 mM) induced contracted jejunum with an EC_50_ value of 0.66 (0.49–0.96), 0.39 (0.28–0.44) and 0.17 (0.10–0.21), similar to verapamil. Concentration–response curves of CaCl_2_ could be significantly moved to the right and down in the presence of STR (0.3, 1 mg/mL).

**Discussion and conclusions:** Results suggest the presence of antidiarrheal activity and spasmolytic effects of STR, possibly mediated through Ca^2+^ channel blocking activity, providing the pharmacological basis for its traditional uses in gastrointestinal disorders.

## Introduction

Diarrhea is the major cause of death in children under the age of 5 (Aleem and Janbaz 2018). Diarrhea can be divided into osmotic, secretory, exudative and gastrointestinal motility disorder diarrhea. Diarrhea refers to the increased frequency and watery consistency of stools, often associated with the discharge of mucus or undigested food. With respect to gastrointestinal motility disorder diarrhea, irritable bowel syndrome (IBS) is a chronic recurrent disease which exists in the general population. Current medical treatments are usually insufficient for patients with chronic IBS. Traditional Chinese medicine (TCM) has been proposed as a promising treatment approach for IBS, and thus has also in recent years been favored for the treatment of diarrhea (Tian 2016).

*Sophora tonkinensis* radix et rhizoma is the dry rhizome of *Sophora tonkinensis* Gagnep (Fabaceae), the herb is listed in the Chinese Pharmacopeia (Committee of National Pharmacopeia. 2015). The rhizome is rich in polysaccharides, alkaloids, flavonoids and other pharmacologically-active ingredients. Research has demonstrated that ST possesses many potentially useful pharmacological effects such as antitumor (An et al. 2016; Li et al. 2016), anti-inflammatory (Chae et al. 2016), antibacterial (Dai et al. 2012), hypoglycemic (Huang et al. 2016), antiviral (Pan et al. 2015) etc. Although ST has many pharmacological activities, research into potential antidiarrheal and spasmolytic activities are scarce. In this study, the antidiarrheal activity of STR was assessed in castor oil-induced diarrheal mice orally. The effect of STR on intestinal contractions and their relationships with Ca^2+^ influx was also investigated to provide further insight into the pharmacological mechanism.

## Materials and methods

### Drugs and reagents

All chemicals of research grade were used for experimental work. Sodium bicarbonate, potassium chloride, magnesium sulfate, glucose, sodium dihydrogen phosphate, sodium chloride, calcium chloride, ferric chloride, aluminum chloride and potassium acetate were produced by Chengdu Cologne Chemicals Co. Ltd. (Chengdu, China). Acetylcholine chloride was from Chengdu Huaxia Chemical Testing Co. Ltd. (Chengdu, China). The castor oil was from Henan Hualong Pharmaceutical Co., Ltd. (Henan, China). Verapamil was from MedChemexpress Co., Ltd. (NJ, USA). Loperamide was from Sigma Chemical Co. (St. Louis, MO, USA). Whereas hydrochloric acid (School of Pharmacy Laboratory Supplies, Nanchong, China), ferric chloride, methanol, potassium acetate and aluminum chloride were used in phytochemical analysis of crude extract. Distilled water was used for the preparation of standard solutions, dilution and physiological salt solutions (Tyrode’s solutions).

### Plant material and preparation

STR was provided by Chengdu Hestia Pharmacy INC and identified by Xue Mei from School of Pharmacy, North Sichuan Medical College and voucher specimen (CBY-2016-0002) was deposited in the herbarium of the same institution. STR was dried in an electric oven at constant temperature (60 °C), and pulverized into a coarse powder (shredding machine: FW177, Taisite, Tianjin, China). Powder (50g) was placed in a 1-L round-bottom flask with seven times the measured mass of methanol as a solvent. Extraction was performed three times under reflux, followed by combination of the filtrate and recovery the solvent. The liquid was concentrated into an extract, and dried in a vacuum decompression drying oven (ZK 6050B, Opson, Wuhan, China). The yield of crude extract of STR was 21%.

### Animals

Adult male Kun Ming mice weighing 18–22 g and locally bred rabbits weighing 2.0–2.5 kg (License No. SYXK (Chuan)-2018-076) were supplied by the Animal Laboratory Center of North Sichuan Medical College (Sichuan, China). The light and dark cycle was maintained for 12 h (temperature 23–26 °C, humidity 70 ± 5%) in the environmental control breeding room for 7 d. White wood chips were used as bedding while animals had free access to water, but fasted for 24 h before the experiments. The animal study was in accordance with the requirements of Institutional Animal Care and the Chinese Commission and followed the required animal welfare and experimental practices.

## Phytochemical study

### Identification of STR extract

#### Preparation of standard solution

Appropriate amount of gallic acid, protocatechuic acid and trifolirhizin were accurately weighed, using methanol (analytical grade) as solvent, to prepare a reference solution including 0.200 mg/mL gallic acid, 0.306 mg/mL of protocatechuic acid, 0.505 mg/mL trifolirhizin, respectively. Then 0.1 mL of each of the above reference solutions were put into the tube, mixed thoroughly. The solution was passed through a 0.22-μm nylon microporous membrane, and kept at 4 °C before use.

#### Preparation of the sample solution

Appropriate amount of STR extract methanol extract (4.236 g/g DW) was accurately weighed to prepare a solution of 52.42 mg/mL of STR, with methanol (analytical grade) as the solvent. The solution was filtered using 0.22-μm nylon microporous membrane, and then used for HPLC analysis.

#### Chromatographic conditions

The standards and samples were analyzed by an Agilent-1220 high performance liquid chromatograph system (Agilent, American). The column was Aglient-ZORBAX SB-C18 (250 mm × 4.6 mm, 5 μm). 0.1% formic acid was used as mobile phase A, Chromatographic acetonitrile was used as mobile phase B. Then the mobile phase was filtered by passing through a 0.45-µm filter membrane. The column loaded with these compounds was run gradiently with a mobile phase consisting of 0.1% formic acid and acetonitrile (cf. Table 1) for the determination of the polyphenols and flavones from STR. The detection wavelength was 260 nm and 310 nm (wavelength switched at 20 min), and the flow rate was 0.6 mL/min. The column temperature was 27 °C, and a sample of 5 μL of this solution was directly injected.

**Table 1.** Liquid chromatography conditions.

Time (min)	Phase A (%)	Phase B (%)
0–10	80	20
10–30	40	60
30–50	80	20

The detection wavelength was switched to 310 nm from 260 nm at 20 min.

### *In vivo* study

#### Acute oral toxicity test

The research of LD_50_ followed the method of Karber (Chen 2011; Gong et al. 2017) with slight modification. Thirty-six male mice with weights between 18 and 22 g were selected and randomly divided into six groups of six mice each. 500, 1000, 2000, 4000, 8000, 16,000 mg/kg body weight (4.19 g crude drug/g) of STR were provided orally to test group. Any signs of toxicity and death were strictly recorded 14 d after administration. During these days, mice had free to access water and food. A dose–response curve was established to determine the LD_50_. The safety of STR was assessed using the single and maximum dose (Chen 2011).

#### Castor oil-induced diarrhea

Referring to the method of Gong et al. (2017) and Guo et al. (2014a), this study first conducted a preliminary experiment. The mice were screened by giving 0.4 mL castor oil and those presenting with diarrhea were randomly divided into five groups of 50 mice each. The negative control group was treated with 0.2 mL normal saline (20 mg/kg) while the positive control group was treated with 0.2 mL loperamide (4 mg/kg). Three test groups were provided orally with STR (125, 250 and 500 mg/kg). Each mouse was caged individually and blotting paper placed under the cage. After 0.5 h of treatment, castor oil (20 mL/kg) was provided orally and the subsequent onset of castor oil-induced diarrhea was observed. The amount of solid feces, semi-solid feces, liquid feces and the time of initial semi-solid appearance within 4 h after castor oil was recorded. The following formula was used to evaluate the severity of diarrhea. Evacuation Index (EI) = solid feces × 1 + semi-solid feces × 2+ liquid feces × 3 (the distribution was as follows: 1 referred to solid feces, 2 referred to semi-solid feces, and 3 referred to liquid feces).

### *In vitro* study

#### Tissue preparation

Healthy rabbits were selected. The animals were provided with water *ad libitum* but underwent fasting for 24 h prior to experimentation. The rabbits were sacrificed by cranial impact. The jejunum was isolated, flushed and put in 4 °C Tyrode’s solution. The jejunum was cut into 2 cm, and mounted vertically in 20 mL organ bath containing Tyrode’s solution maintained at (37 ± 0.5 °C) with a mixture of 95% O_2_ and 5% CO_2_ aerated. After preloading 1 g, the tissue was allowed to equilibrate for 20 min before adding the active compound. Intestinal activity was measured with a force transducer and recorded by BL-420F Physiological Signal Collection and Handing System (Chengdu, China).

#### Effect of STR on spontaneous contraction of rabbit jejunum

After an equilibrium period of 20 min in Tyrode’s solution, cumulative concentrations of STR (0.01, 0.03, 0.1, 0.3, 1, 3, 10 mg/mL) and vehicle were added to determine the effect on spontaneous contraction. To investigate the spasmolytic activity of STR, such as Ca^2+^ antagonistic, Ach (10^−5 ^M) and K^+^ (60 mM) were used as spasmogenic agents. The concentration-dependent inhibitory responses were recorded by adding the test compound in cumulative manner (Mehmood et al. 2011; Janbaz et al. 2012). This method confirms either the spasmolytic action of the test material because of Ca^2+^ channel antagonism, K^+^ channel activation.

#### Effect of STR on CaCl_2_-induced cumulative contractions

The isolated preparations were stabilized in Tyrode’ s solution and were then incubated with Ca^2+^-free high- K^+^(60 mM) solution containing EDTA (0.1 mM) for 0.5 h in order to remove Ca^2+^ from tissue, followed by a Ca^2+^-free high-K^+^ (60 mM) for 15 min. Samples were then treated with the absence and in the presence of STR (0.3, 1 mg/mL) and verapamil (0.3, 1 μM), Ca^2+^ was added in a cumulative fashion (3 × 10^−5^-3 × 10^−2 ^M) to obtain concentration-response curves of CaCl_2_. The contraction induced by 3 × 10^−2 ^M CaCl_2_ in the absence of STR and verapamil was regarded as 100% (Wang et al. 2006).

#### Data analysis

Being represented as mean ± standard error (SEM), all data were analyzed by single-line statistical significance variance analysis (ANOVA) followed by the Dunnett’s test. SPSS 19.0 system was used for testing. *p* ≤ 0.05 was considered statistically significant.

## Results

### Phytochemical study

#### Polyphenols and flavones of STR extract

**Figure 1.**
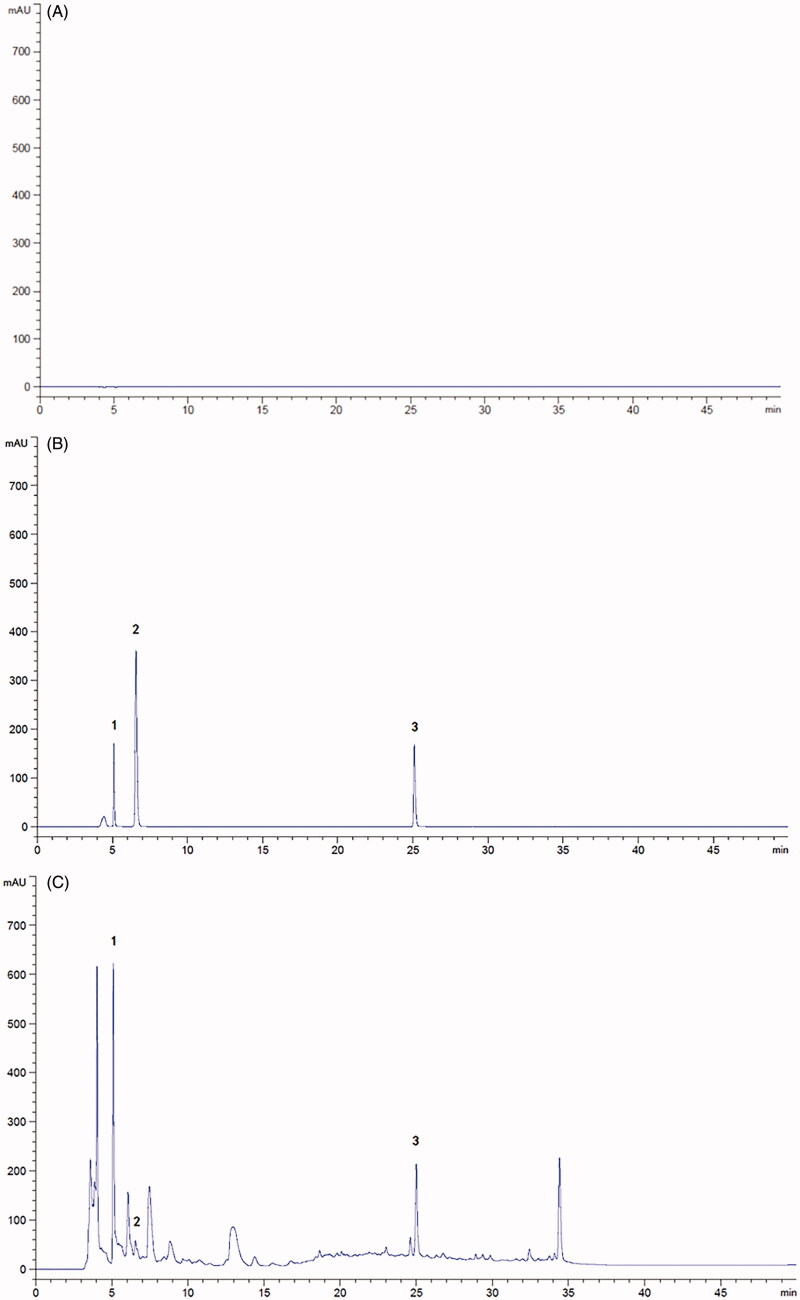
HPLC chromatograms of blank (A), reference substances (B) and the methanol extract of STR (C) (**1** gallic acid, **2** protocatechuic acid, **3** trifolirhizin). A. HPLC chromatogram of the blank. B. HPLC chromatogram of the mixed standards. C. HPLC chromatogram of the STR extract.

Under the optimal liquid chromatography conditions, the separation between gallic acid, protocatechuic acid and trifolirhizin peaks was excellent. The reference substance and the test sample liquid chromatography profiles are shown in Figure 1. The reference solution (Figure 1(B)) and the sample solution (Figure 1(C)) had corresponding chromatographic peaks at the same retention time. Figure 1(C) illustrates the chromatograms obtained from the extract of STR.

### *In vivo* study

#### Acute toxicity test

In the LD_50_ test, there were no signs of death or toxicity in the observation period of ST given in the graded dose of intra-gastric administration. In the single maximum dose test, there were no deaths or changes in physical behavior during the observation period. Based on these results, the LD_50_ value was estimated to be greater than 16,000 mg/kg.

#### Castor oil-induced diarrhea

**Figure 2.**
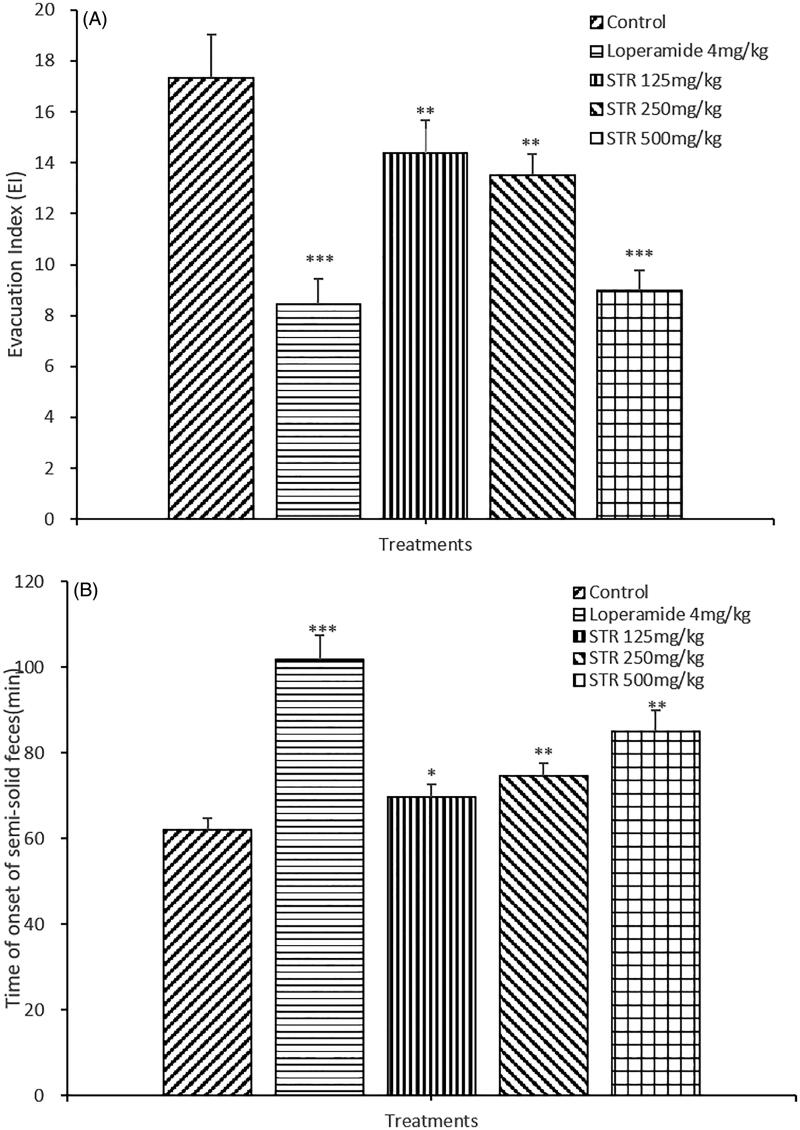
Effect of STR on castor oil-induced diarrhea in mice. A. Evacuation Index (EI), B. Time of onset of semi-solid feces (min). *p < 0.05, **p < 0.01, ***p < 0.001, *n* = 10, compared with the negative control group; volumes presented as the mean ± SEM.

As shown in Figure 2(A), the EI score was found to be 17.35 ± 1.69 in the negative control group. In the 4 h after castor oil administration, all the mice in the negative control group produced liquid feces, with a semi-solid feces onset time of 62 ± 2.7 min (Figure 2(B)). Compared with the negative control group, STR (125, 250 and 500 mg/kg) significantly inhibited castor oil-induced diarrhea, followed by descending the EI score to 14.4 ± 1.26, 13.5 ± 0.85, 9 ± 0.78 (*p* < 0.05 or *p* < 0.01) and ascending the onset time of semi-solid feces to 85.00 ± 4.88, 74.50 ± 3.06, 69.70 ± 2.9 min (*p* < 0.05 or *p* < 0.01), respectively. In addition, there was similarity between STR (500 mg/kg) and loperamide (4 mg/kg), for which the EI was 8.46 ± 0.97 (Figure 2(A)).

#### Effect of STR on spontaneous contraction of rabbit jejunum

**Figure 3.**
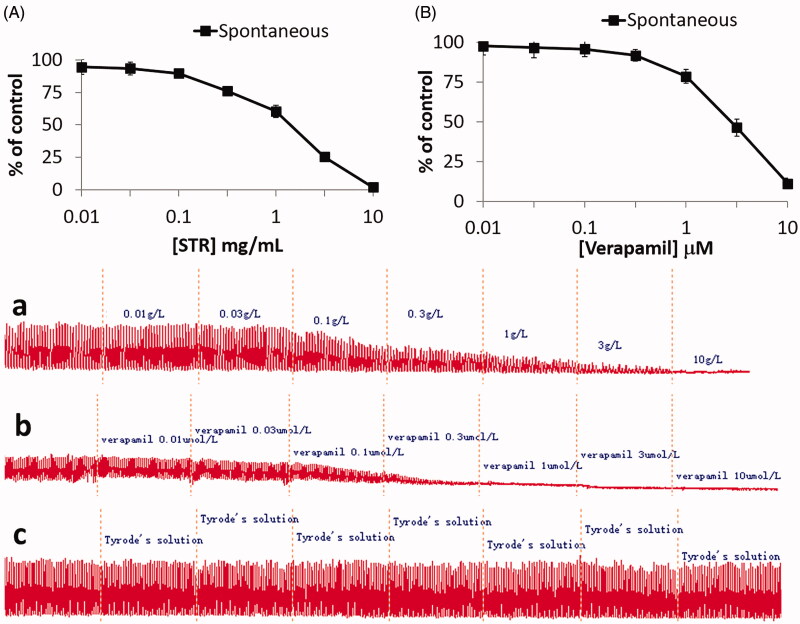
Concentration-dependent inhibitory effect of (A(a)) STR and (B(b)) verapamil, on spontaneously contracting isolated jejunum. Tracing showing (c) spontaneous contraction of isolated rabbit jejunum. Results are expressed as mean ± SEM, *n* = 6.

**Figure 4.**
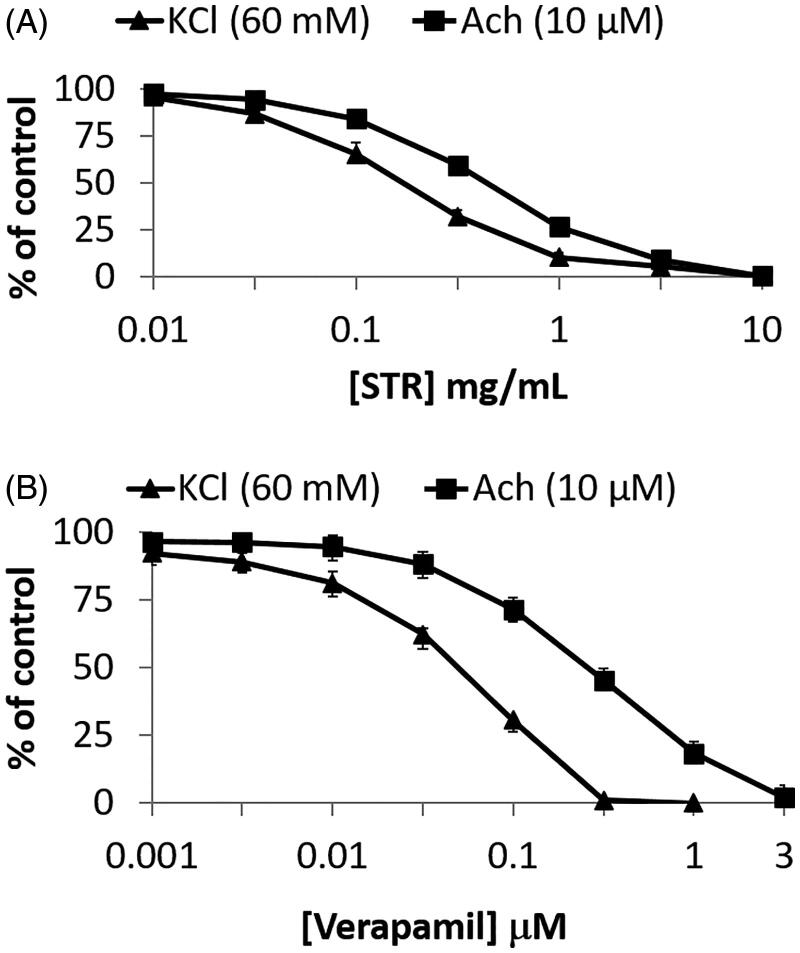
Concentration-dependent inhibitory effect of (A) crude extract of STR and (B) verapamil on high Ach (10^−5 ^M) and K^+^ (60 mM) Ach (10^−5 ^M) induced pre-contracted isolated jejunum. Results are expressed as mean ± SEM, *n* = 6.

STR (0.01–10 mg/mL) inhibited the spontaneous contraction of rabbit jejunum in a concentration-dependent manner with an EC_50_ value of 0.66 mg/mL (0.49–0.96 mg/mL, CI = 95%, *n* = 6) (Figure 3A(a)), similar to verapamil (0.01–10 μM) (Figure 3B(b)) with an EC_50_ value of 0.51 μM (0.42–0.59, 95% CI, *n* = 6). Whereas Ach (10^−5 ^M) and K^+^ (60 mM)-induced contraction in isolated rabbit jejunum was fully relaxed with an increasing concentration of STR at 10 mg/mL, with respective EC_50_ value of 0.39 mg/mL (0.28–0.44 mg/mL, 95% CI, *n* = 6) and 0.17 mg/mL (0.10–0.21 mg/mL, 95% CI, *n* = 6) (Figure 4(A)), similar to verapamil at 0.001–3 μM with an EC_50_ value of 0.25 μM (0.22–0.29, 95% CI, *n* = 6) and 0.041 μM (0.033–0.047, 95% CI, *n* = 6), respectively (Figure 4(B)).

#### Effect of STR on CaCl_2_-induced cumulative contractions

**Figure 5.**
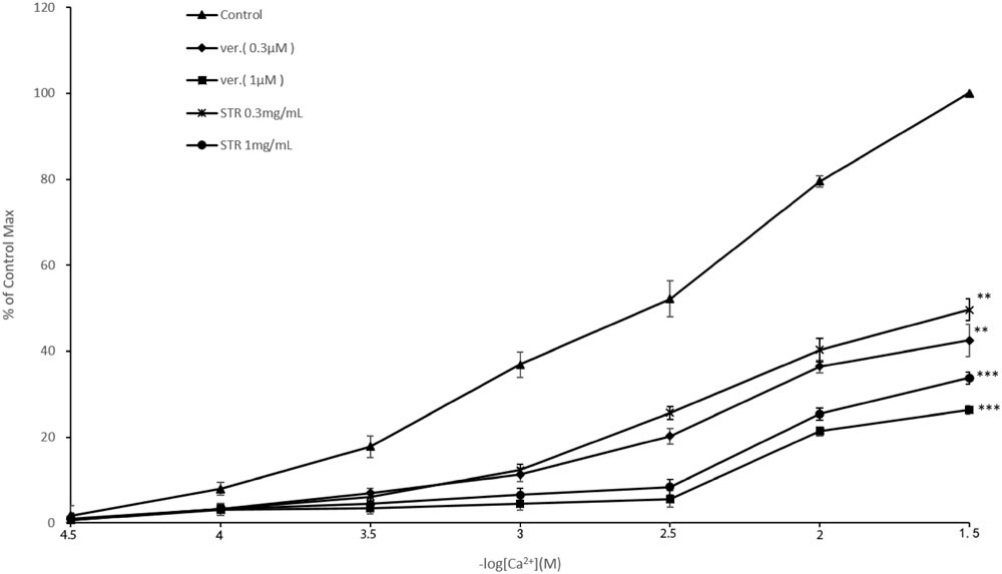
Concentration-response curves of CaCl_2_ on rabbit-isolated jejunum in the absence (▲) and in the presence of STR (× 0.3 mg/mL; • 1 mg/mL) and verapamil (◆ 0.3 μM; ■ 1 μM). Results are mean ± SEM, *n* = 6.

The study indicated STR (0.3, 1 mg/mL) in a concentration-dependent manner noncompetitively antagonized the contraction of isolated tissue preparations induced by cumulative concentration of CaCl_2_. Similarly, in the case of verapamil (0.3, 1 μM), concentration-response curves of CaCl_2_ could be significantly moved to the right and down in the presence of STR (0.3, 1 mg/mL). Compared with the control group, STR (0.3, 1 mg/mL) and verapamil (0.3, 1 μM) reduced the maximum contraction induced by 3 × 10^−2 ^M CaCl_2_ to 33.75 ± 2.53%, 49.66 ± 1.42%, 26.32 ± 0.99% and 42.52 ± 3.71% (*p* < 0.01 or *p* < 0.001), respectively (Figure 5).

## Discussion

Diarrhea is a common gastrointestinal disease with many differing causes. Diarrhea associated with irritable bowel syndrome (IBS) is one of the most common forms occurring in young adults, a disease which seriously affects quality of life and working conditions. IBS is characterized by abdominal pain or discomfort and is often accompanied by abnormal defecation. The disease is common, affecting 5–20% of the general population worldwide (Brandt et al. 2009; Liu and Hou 2011) and 4.6–6% of the population in China (Zhao et al. 2010; Zhang et al. 2014). At present, the pathogenesis of diarrheal-type IBS (IBS-D) remains unclear, with no effective western medicine having been found for the long-term treatment of IBS-D (Bian et al. 2015). It is easy to relapse after drug withdrawal, with serious side effects and with harm to the physical and mental health of the patient (Han et al. 2018). Therefore, looking for Chinese medicine for IBS treatment is a new research direction.

In China, many plants have been found to be effective against treating diarrhea and dysentery; these are commonly used by local people and as part of traditional folk medicine (Zhu et al. 2005; Huang et al. 2010; Guo et al. 2014b). ST, the Chinese traditional herbal medicine, has been documented in many historical texts with respect to its anti-inflammatory and antitumor pharmacological effects. Further studies have shown relevance to gastric disorders *in vitro* bacteriostatic testing showed that STR exhibited a bacteriostatic effect on *Bacillus coli* and *Staphylococcus aureus*, while the experimental results obtained by Yoo et al. (2017) also indicated that SKI3301 in ST can alleviate the spasm of tracheal smooth muscle.

In this acute toxicity test, no death or toxic reaction was observed in mice even after the maximum dosage of 16,000 mg/kg. According to Lorke (1983), any substance without toxic effects at concentrations of 5 g/kg can be considered as relatively safe. As such the STR results indicate an excellent safety profile in these preclinical experiments. This demonstrated the safety profile of the STR and provides a basis for follow-up pharmacological studies *in vivo* (Gong et al. 2017).

Castor oil is a colorless or very pale yellow liquid with a unique flavor obtained from the seeds of castor oil plants. As a plant oil, castor oil has several advantages for pharmacological use, including antimicrobial and antioxidant properties, low toxicity, low cost and easy availability (Yeganeh and Hojati-Talemi 2007; Valera et al. 2012; Holm et al. 2013; Salles et al. 2015). Ricinoleic acid is an active hydrolytic metabolite of castor oil which can induce diarrhea. It not only creates extensive contractions in the transverse and distal colon by inducing changes in electrolyte and water transport (Aleem and Janbaz 2018), but also produces irritant and inflammatory effects on intestinal mucosa, resulting in the release of several mediators including prostaglandins, nitric oxide, platelet activating factor cAMP and tachykinin (Guo et al. 2014a). Therefore, the castor oil model incorporates both motility and secretory diarrhea (Rouf et al. 2003).

ST contains a number of chemicals with potential pharmacologic actions, predominantly comprising flavonoids, alkaloids and polysaccharides (Dai et al. 2012). From previous studies of other medicinal plant extract, it is known that the presence of tannins, alkaloids, flavonoids, reducing sugar, saponins, sterols and terpenes are strongly linked to the antidiarrheal activity of medicinal plants (Di Carlo et al. 1993; Borrelli et al. 2004). Furthermore, flavonoids are known to have strong antidiarrheal activity because of their ability to reduce intestinal motility and hydroelectricity secretion (Aleem and Janbaz 2018). The chemical composition of ST provide a theoretical basis for studying the antidiarrheal effect. It can be speculated that the antidiarrheal effect possibly occurs via regulation of the water and electrolyte permeability of intestinal mucosa, or alternatively by inhibiting the excessive secretion of luminal contents induced by prostaglandin. In the castor oil-induced diarrheal mice, STR significantly inhibited castor oil-induced diarrhea by descending the EI score and ascending the onset time of semi-solid feces which could be taken as antidiarrheal effect.

Mostly, antidiarrheal drugs play a role in reducing the secretion and/or resulting in reduction of GI smooth muscle propulsion. (Tadesse et al. 2017) To further investigate these possibilities, spontaneous contraction of smooth muscle is mainly regulated by periodic cycles of depolarization and repolarization. Depolarizations are evoked by the fast entry of Ca^2+^ into the cytoplasm via the voltage-dependent Ca^2+^ channels and the release of Ca^2+^ from intracellular stores, required for contractile responses and the maintenance of normal tone (Brading 1981; Grasa et al. 2004). By contrast, relaxation occurs due to the decrease of Ca^2+^ in the cytosol. Gastrointestinal contractions are also regulated by a variety of physiological mediators such as histamine, Ach, Serotonin (5-HT) and prostaglandin. Isolated rabbit jejunum smooth muscle was used as a model for further exploration of mechanism of action in gastrointestinal smooth muscle. Intestinal smooth muscle induced by acetylcholine was treated with different concentrations of STR. The results showed that STR inhibited acetylcholine-induced contraction in a concentration-dependent manner. Based on this, it can be hypothesized that the inhibition effect on smooth muscle contraction induced by STR might be regulated by muscarinic receptors.

Correlating research has shown that K^+^ at high concentrations (> 30 mM) is capable of opening voltage-dependent Ca^2+^ channels (VDCs). This leads to smooth muscle contractions, essentially causing a contractile effect by influx of extracellular Ca^2+^ (Aleem and Janbaz 2018). Substances that inhibit potassium-induced contraction are considered to be Ca^2+^ channel blocker compounds (Godfraind et al. 1986; Gilani et al. 2007). The experimental results showed that STR exhibited a spasmolytic effect on the contraction induced by K^+^ (60 mM). It can be hypothesized that the pathway of action may be related to LL-type calcium channel. To assess the relationship between STR and L-type calcium channel, concentration-response relationship curves of cumulative calcium chloride were determined. Experimental results showed that STR could make the curves of calcium chloride move to the right and down, an effect which was similar to that of verapamil. This further indicated that STR worked as a noncompetitive antagonist of the entry of extracellular calcium ions, which weakened the contraction of smooth muscle.

## Conclusions

The present results suggest the presence of spasmolytic effects in the methanol extract of STR, possibly mediated through Ca^2+^ channel-blocking activity, providing the pharmacological basis for its traditional uses in gastrointestinal disorders.
